# Reorganization of brain networks in olfactory groove meningioma patients: a pilot resting-state fMRI study

**DOI:** 10.3389/fneur.2025.1644138

**Published:** 2025-08-29

**Authors:** Elena Filimonova, Anton Pashkov, Aleksandra Poptsova, Galina Moysak, Azniv Martirosyan, Polina Prozorova, Vladimir Kurilov, Jamil Rzaev

**Affiliations:** ^1^FSBI "Federal Center of Neurosurgery", Novosibirsk, Russia; ^2^Department of Neurosurgery, Novosibirsk State Medical University, Novosibirsk, Russia; ^3^Department of Neuroscience, Institute of Medicine and Psychology, Novosibirsk State University, Novosibirsk, Russia; ^4^Department of Data Collection and Processing Systems, Novosibirsk State Technical University, Novosibirsk, Russia

**Keywords:** meningioma, olfactory groove meningioma, functional MRI, functional connectivity, CONN

## Abstract

**Background:**

Olfactory groove meningioma (OGM) is frequently associated with neuropsychological and behavioral impairments. However, there is currently a lack of evidence on the pathobiology of these functional alterations. In this study, our objective was to evaluate functional connectivity disturbances in patients with OGMs compared to healthy controls.

**Methods:**

Nineteen patients with OGMs and twenty healthy controls were enrolled. The seed-based functional connectivity analysis used the main hubs of the default mode network (DMN), salience network (SN), and fronto-parietal network (FPN) as seeds. Region-of-interest (ROI)-to-voxel second-level analysis was conducted, revealing the most significant clusters of differences in brain functional connectivity between the groups.

**Results:**

Patients with OGMs demonstrated significant alterations in resting-state functional connectivity within the DMN, SN, and FPN compared to controls. Specifically, within the DMN, we identified abnormal connectivity patterns involving the medial prefrontal cortex bilaterally, posterior cingulate cortex bilaterally, and right posterolateral cortex. In the SN, we observed enhanced functional connectivity between the anterior cingulate cortex bilaterally and left frontal, temporal, and insular regions. Additionally, the FPN exhibited disrupted connectivity of the right posterior parietal cortex with other brain areas. Notably, some connectivity changes were related to perilesional edema volume, visual acuity, and clinical metrics (KPS and MoCA scores).

**Conclusion:**

We revealed significant alterations in DMN, SN, and FPN function in patients with olfactory groove meningiomas compared with controls. These changes were associated with clinical variables and lesion characteristics. To our knowledge, this is the first report on rs-fMRI alterations in patients with olfactory groove meningiomas.

## Introduction

Olfactory groove meningiomas (OGMs) represent a relatively uncommon subset of skull base neoplasms, typically benign and slow-growing, comprising approximately 6–18% of all intracranial meningiomas (1). Due to their indolent progression, patients often present at advanced stages, with tumors reaching considerable dimensions before clinical detection. The most frequently reported symptoms include headaches, visual deficits, cognitive impairment, and neuropsychiatric disturbances such as emotional dysregulation or personality changes. Documented psychiatric manifestations include depression (2, 3), apathy (4), visual hallucinations (5), confabulation (6), and executive dysfunction (7) among others. Critically, emerging evidence suggests that some deficits persist postoperatively, with incomplete restoration of premorbid functioning despite successful tumor resection (8).

Brain magnetic resonance imaging (MRI) plays a crucial role in the OGM diagnosis (1), offering comprehensive information on the anatomy of the skull base and anterior fossa to design an appropriate treatment plan (9, 10). It is generally believed that tumor size, peritumoral edema and the degree of mass effect on adjacent brain structures critically influence symptom manifestation (9).

Previous research in this area that has focused on tumor morphometry and demonstrated that larger OGMs (exceeding 3–4 cm in diameter) exhibit a stronger correlation with neuropsychiatric symptomatology (11–13). A positive relationship between mental disturbances and the presence and severity of edema observed in preoperative MRI scans was also indicated (14). However, no published neuroimaging studies have systematically assessed functional brain alterations in this patient group. Nevertheless, chronic perilesional edema and frontal lobe compression may induce widespread functional network disruptions, contributing to systemic cognitive and behavioral deficits.

The brain functional connectivity has been extensively studied due to its essential roles in cognitive functions and emotions, both in healthy controls and the broad spectrum of pathological conditions (15–17). Blood Oxygen Level Dependent functional MRI (BOLD fMRI) has emerged as a powerful and versatile tool for assessing neural activity, offering unique insights into neurophysiological and pathological processes (17). This technique, like other functional neuroimaging modalities, relies on neurovascular coupling principles (18). Resting-state fMRI (rs-fMRI) has gained particular prominence due to its methodological accessibility and ability to evaluate spontaneous neural activity through resting-state networks (RSNs). Multiple RSNs have been identified, with established roles in cognitive and behavioral processes (15, 17, 18). Among RSNs, the default mode network (DMN), frontoparietal network (FPN), and salience network (SN) have been most consistently identified and thoroughly investigated (15, 18). It is believed that DMN, which includes key regions such as the medial prefrontal cortex (mPFC) and posterior cingulate cortex (PCC), is critical for self-referential thinking, memory, and social cognition (19); the FPN, encompassing dorsolateral prefrontal and posterior parietal regions, is essential for executive control, working memory, and attention (20); the SN, anchored in the anterior insula and anterior cingulate cortex, plays a pivotal role in interoception, emotional regulation, as well as switching between the DMN and FPN (21). However, the specific contributions of these networks to behavioral disturbances in patients with OGMs remain insufficiently characterized.

Alterations in the resting-state functional connectivity have been documented in different psychiatric conditions, including major depressive disorder (22), bipolar disorder (23), and schizophrenia (24), as well as chronic traumatic brain injury (25, 26). For example, Liu et al. (27) reported widespread alterations in regional homogeneity of BOLD signal in patients after frontal lobe injury (27). Emerging evidence suggests that olfactory deficits also correlate with altered rs-fMRI functional connectivity (28–30). Additionally, the application of rs-fMRI in neuro-oncology has also expanded in recent years (31, 32). Alterations in brain functional connectivity have been detected in patients with meningioma even after surgical resection (33). It has also been shown that perifocal brain edema in meningioma patients is associated with impaired whole-brain rs-fMRI connectivity (34). Therefore, the analysis of the resting state connectivity could provide valuable information on brain functioning in patients with different pathologies, including OGMs.

To date, there are still limited published data on pathobiology of behavioral alterations in patients with OGMs, and there are no published data on brain functional connectivity alterations in this group. In this study, we focused on examining functional connectivity within the DMN, FPN, and SN in patients with OGMs. The networks were selected based on their well-established roles in cognitive and behavioral functions that could be affected by frontal lobe compression—a hallmark of OGMs. Our objective was to examine variations in functional connectivity within these resting-state networks between patients with OGMs and a control group. We hypothesized that OGMs patients would exhibit brain functional connectivity changes, which would be associated with both tumor and brain edema size. Furthermore, we anticipated that these changes in functional parameters would be linked to clinical symptoms.

## Materials and methods

### Participants

We conducted a prospective observational study using a cross-sectional design. The study involved patients with olfactory groove meningiomas who underwent surgical treatment at our hospital between January 2022 and December 2024, as well as healthy controls matched for age and sex. All participants underwent high-resolution brain MRI scans and comprehensive neurological assessments. A total of 39 participants were enrolled, consisting of 19 patients with olfactory groove meningiomas and 20 controls. The inclusion criteria for patients were: (1) diagnosis of olfactory groove meningioma (morphologically verified); (2) age between 18 and 80 years; and (3) high-quality MRI data. The exclusion criteria for both groups were as follows: (1) multiple meningiomas; (2) significant neurological, psychiatric, cardiovascular, or oncological comorbidities; (3) a history of previous neurosurgical interventions; (4) poor-quality MRI data.

All patients underwent comprehensive preoperative neurological and ophthalmological evaluations. The assessment protocol included the Karnofsky Performance Scale (KPS), visual acuity testing (VAT), and cognitive evaluation using the Montreal Cognitive Assessment (MoCA). Each patient provided written informed consent to participate in the study, which was conducted in accordance with the Declaration of Helsinki and approved by the local Ethics Committee of the Federal Center for Neurosurgery, Novosibirsk, Russia (protocol no. 5 dated 03-14-2023).

### MRI data acquisition

MR imaging data were acquired using a 3 T system (Ingenia, Philips Healthcare, The Netherlands) equipped with a 16-channel receiver head and neck coil. The MRI protocol included conventional MRI sequences (details provided in Supplementary Table 1), as well as resting-state BOLD fMRI, and high-resolution 3D T1-WI before and after contrast injection. Resting-state BOLD functional MRI had the following parameters: TR, 3000 ms; TE, 30 ms; FOV, 240*240 mm; flip angle, 90; matrix, 80*80; 45 axial slices with interleaved acquisition and full brain coverage; slice thickness, 3 mm. Before rs-fMRI session patients were instructed to keep their eyes closed, not to think about anything in particular and not falling asleep. 3D T1-WI (acquired in the sagittal plane) had the following parameters: TR, 6.56 ms; TE, 2.95 ms; FOV, 256*256 mm; flip angle, 8; matrix, 256*256; and slice thickness, 1 mm. The total acquisition time was approximately 35 min.

### Tumor segmentation

For each patient, the olfactory groove meningioma was segmented with ITK-Snap software (version 4.0.0, http://www.itksnap.org) via a semiautomatic classification algorithm. Postcontrast T1-WI, and T2-WI were registered and resampled to T1-WI via SPM12 with a normalized mutual information cost function and 4th-degree B-spline interpolation.^1^ Tumor segmentation was performed on postcontrast T1-weighted images, referred to as T2-weighted images. After that, perilesional edema segmentation was performed (when presented) based on T2-WI. The segmentation results were saved as binary masks in NifTI format with subsequent volume calculation.

### Resting-state fMRI data processing

At first, the acquired structural and functional MR images were subjected to a quality assessment via the MRI-QC package.^2^ Following rs-fMRI data processing was performed using the CONN toolbox version 20b.^3^ Functional images were preprocessed and denoised using the default options in CONN (35). In all cases, the preprocessing and artifacts correction results were visually verified by a certified neuroradiologist. Additionally, individual tumor and perilesional edema volume values, as well as clinical and demographic data were included as covariates for subsequent analysis. Second-level functional connectivity analysis was performed using seed-based algorithm (15). Since the study aimed to assess functional connectivity in the DMN, FPN, and SN, we selected the following seed regions: the medial prefrontal cortex (mPFC), lateral prefrontal cortex (lPFC), posterior cingulate cortex (PCC), posterolateral cortex (PL), posterior parietal cortex (PPC), supramarginal gyrus (SMG), and anterior insula (AI). An illustration of the data processing procedure is shown in Supplementary Figure 1.

### Statistical analysis

Descriptive statistic was performed using R software.^4^ Demographic and clinical data were non-normally distributed (Shapiro–Wilk test). Cluster-based permutation/randomization analysis of functional connectivity differences between groups was performed using the CONN v20b internal procedure (35). This approach works as follows: (1) cluster formation: brain regions showing connectivity differences between groups are grouped into spatially contiguous “clusters” based on their statistical strength; (2) permutation testing: to determine whether these clusters reflect true group differences (rather than random chance), the group labels (patient versus control) are randomly shuffled thousands of times, creating a null distribution of cluster sizes; (3) significance thresholding: only clusters larger than 95% of those in the null distribution are considered statistically significant. An assessment of the effects of clinical and morphometric variables—such as tumor and edema volumes, MoCA and KPS scores, and VAT—on functional connectivity was also performed within the OGM group using a general linear model (GLM). A *p* value less than 0.05 was regarded as statistically significant, after Family-Wise Error (FWE) correction.

## Results

### Clinical and demographic data

Nineteen individuals with OGMs (4 men and 15 women, 37 to 70 years of age with an average age of 58.7 years) and 20 healthy controls (4 men and 16 women, 41 to 74 years of age with an average age of 57.8 years) were included in the study. Table 1 presents the clinical and demographic details of the participants. There were no significant differences between the PTN and control groups in terms of age (*p* > 0.1) or sex (*p* > 0.1). There were 17 patients with Grade 1 tumors, and 2 with Grade 2 tumors. The tumor volume varied from 7.75 mL to 194.3 mL (*M* = 35.5 mL, IQR = 40.06 mL). The volume of perilesional edema ranged from 0 mL (no edema) to 141.2 mL (*M* = 30.39 mL, IQR = 78.3 mL).

**Table 1:** Participants’ characteristics.

	OGMs group (*n* = 19)	Control group (*n* = 20)	*p*-value
Gender (M:F ratio)	0.26: 1	0.25: 1	*p* > 0.1
Age (years)	58.7 ± 8.3	57.8 ± 6.9	*p* > 0.1
Tumor volume (mL) M (IQR)	35.5 (40.06)	NA	–
Peritumoral edema (mL) M (IQR)	30.39 (78.3)	NA	–
MoCA score M (IQR)	18 (5)	NA	–
KPS score M (IQR)	70 (20)	NA	–
VAT M (IQR)	0.7 (0.79)	NA	–

OGMs, olfactory groove meningiomas; MoCA, Monreal cognitive assessment scale; KPS, Karnofsky performance scale; VAT, visual acuity test; IQR, interquartile range; NA, non applicable.

### Differences in the DMN, FPN, and SN functional connectivity between the OGMs and control groups

**Figure 1:**
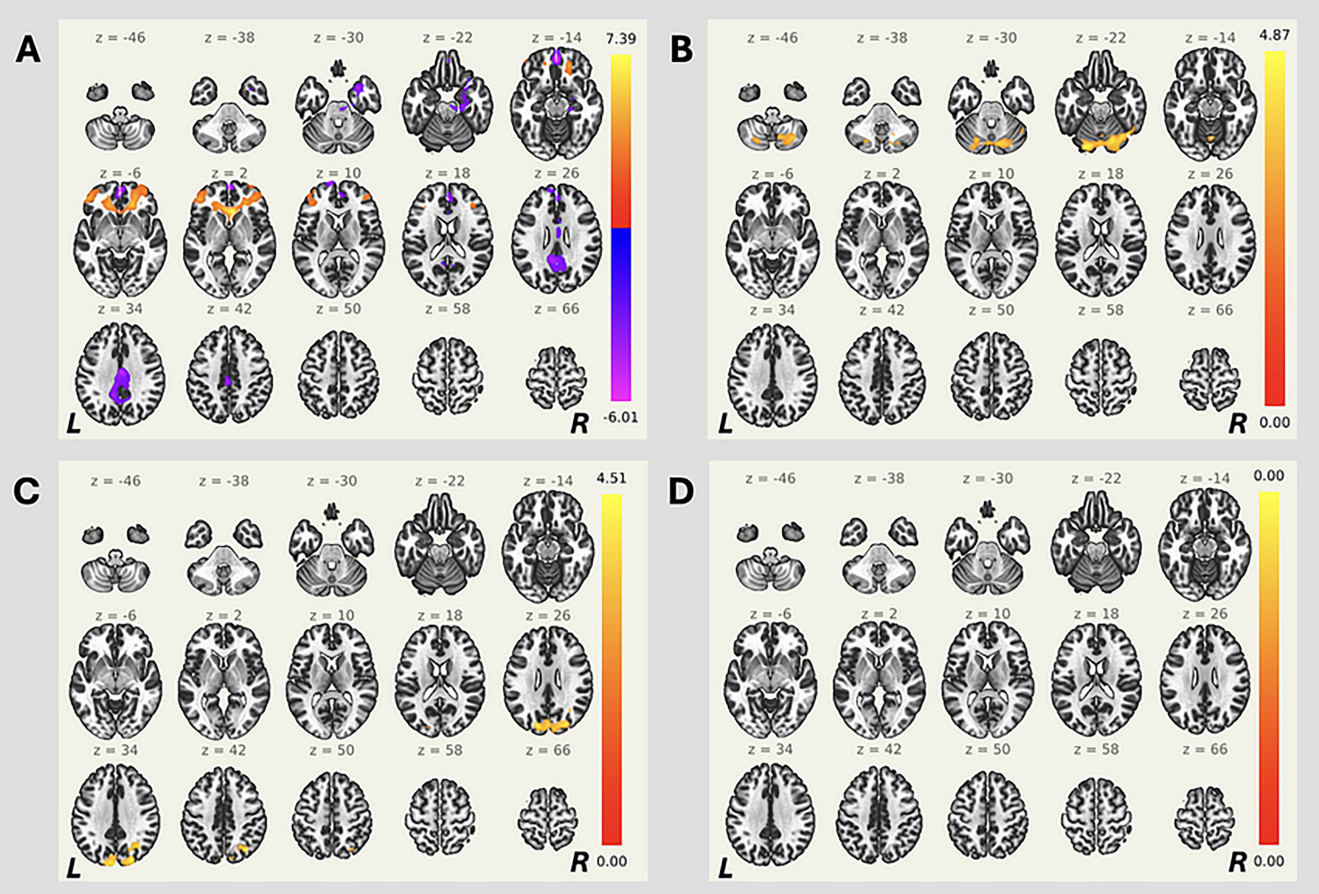
Seed-based resting-state functional connectivity differences between patients with OGMs and controls. Cluster-based permutation testing was performed (GLM contrast: OGM > controls). Seed regions: **(A)** MPFC, **(B)** PCC, and **(C)** right PL. No significant differences were observed when seeding from **(D)** left PL. Side bars represent *T*-statistic; clusters thresholded at *p* < 0.05 (FWE-corrected).

Patients with OGMs exhibited reduced functional connectivity between the MPFC and such regions as the bilateral posterior cingulate gyrus and precuneus, compared to controls (all p-FWE < 0.05; Table 2; Figure 1A), as well as enhanced functional connectivity between the MPFC and different frontal regions bilaterally. Furthermore, patients with OGMs demonstrated increased functional connectivity between the PCC and bilateral cerebellar hemispheres (p-FWE = 0.02; Table 2; Figure 1B). The analysis also revealed enhanced functional connectivity of the right PL with bilateral occipital cortices in the OGMs group (p-FWE = 0.03; Table 2; Figure 1C). No statistically significant differences between groups were detected in the left PL functional connectivity (p-FWE > 0.05, Figure 1D).

**Table 2:** The results of group analysis (OGMs vs. controls).

Seed	Centre of cluster (*x*; *y*; *z*)	Size of cluster (voxels)	*T*-value	p-FWE	Involved anatomical structures
Default Mode Network (DMN)					
MPFC	+4; +26; +2	2,830	+6.4	0.019	Left and right frontal poles, subcallosal cortex, anterior cingulate cortex, right orbitofrontal cortex and middle frontal gyrus
	−8; −44; +30	2,190	−4.5	0.036	cingulate gyri, precuneous bilaterally
PCC	−22; −80; −22	2,447	+4.4	0.022	Right and left cerebellar hemispheres
right PL	+34; −66; +42	1823	+4.1	0.031	Right and left occipital poles, cuneus and precuneous bilaterally
Fronto-Parietal Network (FPN)					
right PPC	+12; +34; −24	1,569	+ 4.3	0.035	Right and left orbitofrontal cortex, subcallosal cortex, right and left frontal pole, medial frontal cortex, left inferior frontal gyrus and temporal pole
	+18; −46; +76	1,477	−3.2	0.043	Right precentral and postcentral gyri, superior and middle frontal gyri, superior parietal lobule, angular gyrus
	+32; +24; −8	1,425	−2.8	0.049	right frontal pole, orbitofrontal cortex, insula
Salience network (SN)					
ACC	−44; +16; +12	2,449	+4.6	0.028	Left opercular cortex, inferior frontal gyrus, superior and middle temporal gyri, Heschl’s gyrus, temporal pole, supramarginal gyrus, and precentral gyrus

OGMs, olfactory groove meningiomas; MPFC, medial prefrontal cortex; PCC, posterior cingulate cortex; PL, posterolateral cortex; PPC, posterior parietal cortex; ACC, anterior cingulate cortex.

**Figure 2:**
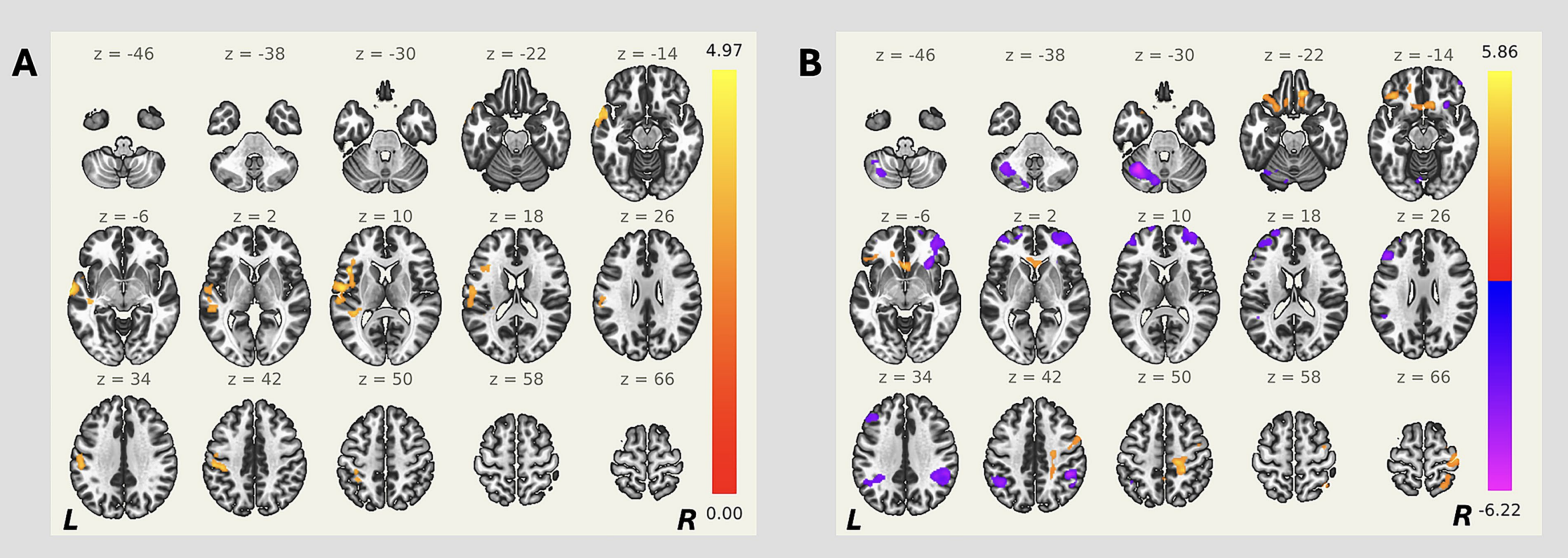
Seed-based resting-state functional connectivity differences between patients with OGMs and controls. Cluster-based permutation testing was performed (GLM contrast: OGM > controls). Seed regions: **(A)** ACC, and **(B)** right PPC. Side bars represent *T*-statistic; clusters thresholded at *p* < 0.05 (FWE-corrected).

Patients with OGMs also demonstrated enhanced functional connectivity between the ACC and such regions as the left insular and opercular areas, pre- and postcentral gyri, as well as temporal cortex, compared to controls (p-FWE < 0.05; Table 2; Figure 2A). No statistically significant differences between groups were detected in functional connectivity of other hubs of the salience network (all p-FWE > 0.05, not shown).

The analysis revealed enhanced functional connectivity of the right PPC with bilateral frontal poles and orbitofrontal cortices in the OGMs group, as well as decreased functional connectivity of the right PPC with other frontal regions bilaterally in the OGMs group (all p-FWE < 0.05; Table 2; Figure 2B). No statistically significant differences between groups were detected in functional connectivity of other hubs of the fronto-parietal network (all p-FWE > 0.05, not shown).

### Effects of the tumor and perilesional edema volume on functional connectivity data

We revealed a positive impact of peritumoral edema volume on the functional connectivity of the right anterior insula region with the posterior cingulate and precuneus cortices bilaterally (p-FWE = 0.023, Supplementary Table 2). No other significant effects were detected among the remaining seed regions (all *p* > 0.05, not shown). Tumor volume did not significantly affect functional connectivity in any of the predefined seed regions (all *p* > 0.05, data not shown).

### Effects of the clinical scores on functional connectivity data

We found a statistically significant negative effect of KPS score on functional connectivity between the left PL and bilateral frontal cortex (p-FWE = 0.031, Supplementary Table 2). Negative effect of KPS score on functional connectivity between the right PPC and the right occipital cortex was also identified (p-FWE = 0.003, Supplementary Table 2). Additionally, we found positive association between KPS score and functional connectivity of the left anterior insula with the right insular and opercular cortices (p-FWE = 0.017, Supplementary Table 2). Furthermore, KPS scores positively affected functional connectivity between the right anterior insula and bilateral cingulate cortex (p-FWE = 0.013, Supplementary Table 2). No other significant impacts were detected among the remaining seed regions (all *p* > 0.05, not shown).

We also detected a statistically significant negative effect of MoCA scores on MPFC—right frontal cortex functional connectivity (p-FWE = 0.008, Supplementary Table 2). Positive effect of MoCA scores on left PL—right precentral cortex functional connectivity was also identified (p-FWE = 0.02, Supplementary Table 2). Additionally, we found negative association between MoCA score and functional connectivity of the left PPC with the right lingual and fusiform cortices (p-FWE = 0.03, Supplementary Table 2). Furthermore, MoCA scores showed a positive effect on functional connectivity between the right PPC and right occipital cortex (p-FWE = 0.002, Supplementary Table 2). No other significant impacts were detected among the remaining seed regions (all *p* > 0.05, not shown).

Additionally, we observed a statistically significant positive association between visual acuity and functional connectivity of the MPFC with the bilateral visual cortex (p-FWE = 0.038, Supplementary Table 2). Furthermore, visual acuity showed positive effect on functional connectivity between the left SMG and bilateral frontal lobes (p-FWE = 0.029, Supplementary Table 2). No other significant impacts were detected among the remaining seed regions (all p > 0.05, not shown).

## Discussion

Assessing functional brain impairments in patients with CNS tumors represents a crucial research focus in this field. This study specifically examined patients with OGMs due to their unique anatomical location involving progressive frontal lobe compression and frequent cognitive/behavioral manifestations. We compared functional connectivity between key nodes of major brain networks (DMN, SN, and FPN) and various brain regions in OGM patients versus controls. To our knowledge, this is the first study to investigate complex functional connectivity alterations in OGM patients using resting-state BOLD fMRI. Our findings support the hypothesis that cognitive and behavioral changes in OGM patients may be associated with disrupted neuronal network functioning.

We identified significant functional connectivity changes affecting different DMN hubs in OGMs patients. Specifically, we observed altered connectivity patterns in such regions as MPFC, PCC, and right PL. The DMN, being the most extensively characterized resting-state network, demonstrates well-documented functional disturbances across various neuropsychiatric conditions including major depressive disorder (36), bipolar disorder (37), and schizophrenia (37). In our OGM cohort, these alterations likely stem from direct mechanical compression of frontal lobe structures (particularly the MPFC) by the tumor, as well as the secondary effects of peritumoral edema. These pathological effects appear to disrupt normal DMN integrity, potentially triggering the formation of compensatory neural connections as an adaptive response.

We also found an enhanced functional connectivity between the ACC and multiple regions—including the left insular/opercular areas, precentral and postcentral gyri, and temporal cortex—in OGM patients compared to controls. As a key hub of the salience network, the ACC maintains extensive structural and functional connections with frontal lobe regions (38), supporting its critical role in emotional and behavioral regulation. Alterations in ACC functional connectivity have been described in several neuropsychiatric conditions, including chronic insomnia (39), major depressive disorder (40, 41), and borderline personality disorder (38). Several studies have reported increased functional connectivity between the anterior cingulate cortex (ACC) and other brain regions (38, 39), while others have observed decreased connectivity (40). These divergent findngs highlight the pathobiological complexity across different neuropsychiatric conditions. While our study provides preliminary evidence regarding ACC connectivity alterations in OGM patients, further research is needed to validate these findings and elucidate the precise role of ACC functional connectivity changes in this population.

Furthermore, our analysis identified altered functional connectivity between the right PPC and the bilateral frontal regions. PPC is known to participate in visuomotor integration (45) and visual search processes (46). Thus, revealed connectivity changes are particularly relevant given the frequent presentation of visual disturbances in patients with OGMs (42), suggesting potential neuroplasticity activation due to tumor-related visual pathway disruption. Enhanced fronto-parietal functional connectivity may maintain visuospatial function despite tumor mass effects on primary visual pathways, consistent with reports of neuroplasticity in other slow-growing tumors (32). Alternatively, increased connectivity might mirror maladaptive over-recruitment of attentional networks, potentially contributing to the visual disturbances frequently reported in OGM patients (42). Future studies combining longitudinal fMRI with detailed neuro-ophthalmological assessment could clarify whether these findings predict clinical recovery or progressive function loss.

In our study, associations of the functional connectivity with clinical data were also revealed. Visual acuity showed a positive association with enhanced connectivity of MPFC and left SMG with different regions of the brain. Cognitive performance data (MoCA scores) were associated with a decrease in connectivity between the bilateral PPC and the right occipital regions, a decrease in connectivity between the right frontal regions, and a greater connectivity between the MPFC and the right occipital regions. Finally, KPS scores were linked to altered connectivity patterns in DMN, SN, and MPFC hubs. These findings may reflect distinct network-specific relationships with different clinical domains in OGM patients.

Interestingly, we did not observe effects of tumor volume on changes in functional connectivity. However, previous studies in this field have demonstrated an association between tumor size and neuropsychiatric symptomatology in patients with OGMs (42). This discrepancy could be attributed to our study’s small sample size and consequent low statistical power. Additionally, most patients in our cohort presented with large-sized OGMs at baseline, resulting in a non-normal data distribution that may have further influenced the results.

However, we identified significant associations between peritumoral edema volume and alterations in functional connectivity. Specifically, edema volume showed a positive impact on connectivity between the right anterior insula (a key node of the salience network) and bilateral precuneus/posterior cingulate cortices. In healthy individuals, the anterior insula normally exhibits strong connectivity with the anterior cingulate gyrus (43), supporting cognitive functions (44). However, in OGM patients, peritumoral edema frequently affects the ACC region. The observed positive link between right anterior insula-PCC connectivity and peritumoral edema volume in our cohort may therefore represent compensatory neuroplastic reorganization in response to pathological disruption.

Together, the findings of our study provide evidence of altered functional connectivity in patients with OGMs, which may be associated with progressive compression of the frontal lobes. In addition to the broad functional reorganization detected in OGMs patients, our analysis revealed a correlation between these neural changes and clinical data. This finding suggests that altered functional connectivity may represent neurobiological substrates underlying symptoms pathogenesis. However, the small sample size in the OGMs group prevented us from drawing any profound conclusions except for the preliminary ones. Exploring the importance of functional connectivity abnormalities in the pathogenesis of behavioral disturbances in patients with OGMs, along with their clinical implications, could be a promising avenue for future research.

This study has several limitations. First, the sample size was relatively small (*n* = 19) due to the rarity of this condition; while this enabled preliminary characterization of functional connectivity alterations, larger cohorts are needed to strengthen these findings. Second, the absence of longitudinal follow-up data precludes assessment of postoperative network reorganization; this could be an important direction for future investigation. Finally, the presence of mass lesions and perilesional edema may distort normal neurovascular coupling and anatomical relationships, potentially confounding functional connectivity measurements. Further research with a larger sample size, follow-up data, comprehensive neurological and neuropsychological evaluations, and modern postprocessing techniques is necessary to validate these findings.

## Conclusion

We identified significant functional changes in the DMN, SN, and FPN in patients with olfactory groove meningiomas compared to controls. Moreover, these changes were associated with peritumoral edema volume, as well as clinical findings. To our knowledge, this is the first report on rs-fMRI alterations in patients with olfactory groove meningiomas. These preliminary findings provide new insights into the mechanisms underlying the behavioral disturbances observed in this condition.

## Data Availability

The raw data supporting the conclusions of this article will be made available by the authors, without undue reservation.

## Footnotes

^1^ http://www.fil.ion.ucl.ac.uk/spm/

^2^ https://mriqc.readthedocs.io/en/latest/

^3^ https://web.conn-toolbox.org/

^4^ www.r-project.org

## Supplementary material

SUPPLEMENTARY FIGURE 1 Schematic representation of the MRI data postprocessing algorithm with the ITK-Snap and CONN v20b toolboxes.
