# Supplementary figures and images for: Reorganization of brain networks in olfactory groove meningioma patients: a pilot resting-state fMRI study
Source: Front Neurol. 2025 Aug 29;16:1644138. doi: 10.3389/fneur.2025.1644138 (PMC12425792; doi:10.3389/fneur.2025.1644138)

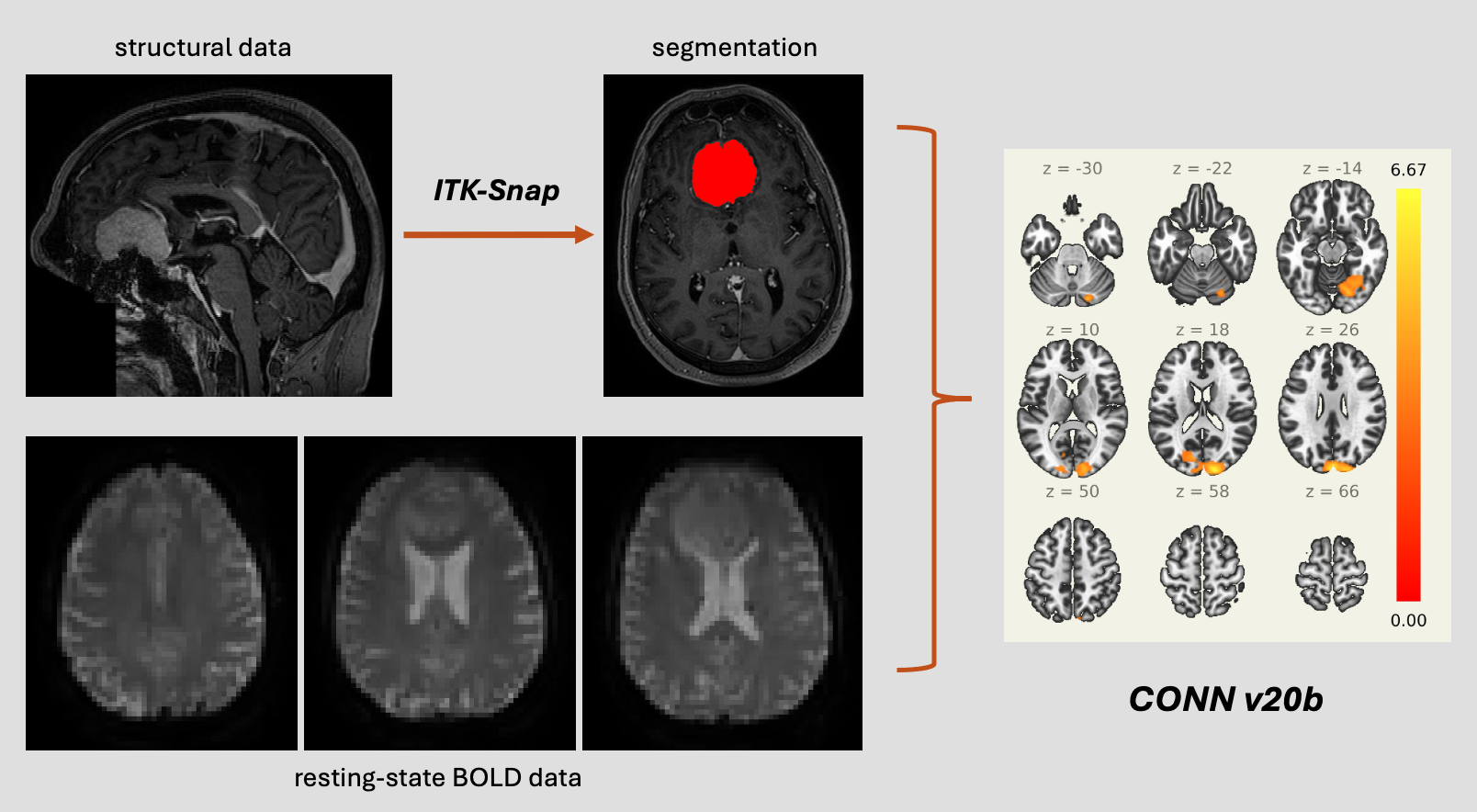

Supplement: SUPPLEMENTARY FIGURE 1 — Schematic representation of the MRI data postprocessing algorithm with the ITK-Snap and CONN v20b toolboxes. [file Image_1.TIFF]
