# Supplementary material for: Reorganization of brain networks in olfactory groove meningioma patients: a pilot resting-state fMRI study
Source: Front Neurol. 2025 Aug 29;16:1644138. doi: 10.3389/fneur.2025.1644138 (PMC12425792; doi:10.3389/fneur.2025.1644138)

**Supplementary file 1. Example of ROIs selection in patient with OGM (CONN software)**

1. Default Mode Network

MPFC at the level of corpus callosum genu


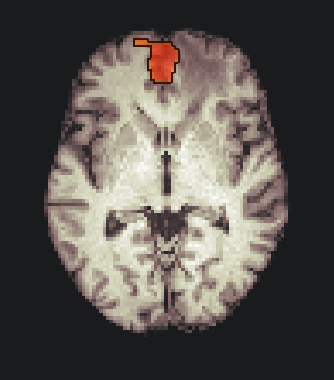


MPFC at the level of basal frontal areas (almost no activation)


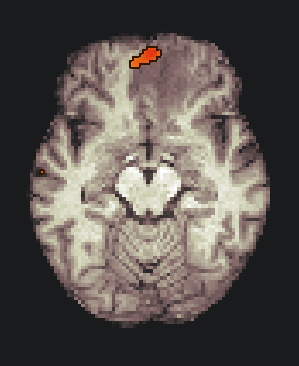


PCC


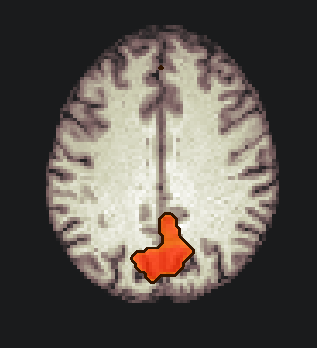


Left PL


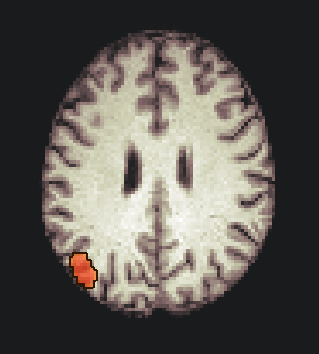


Right PL


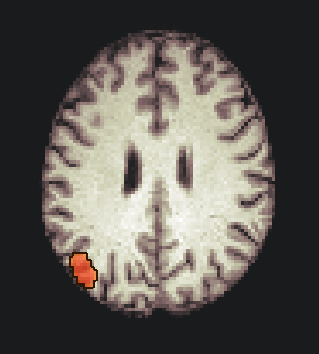


1. Fronto-Parietal Network

Left LPFC


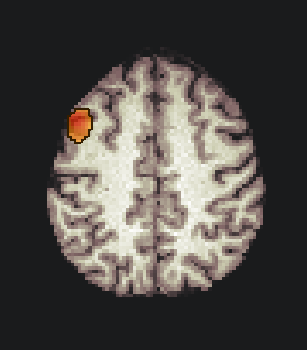


Right LPFC


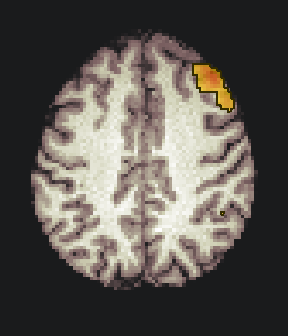


Right PL


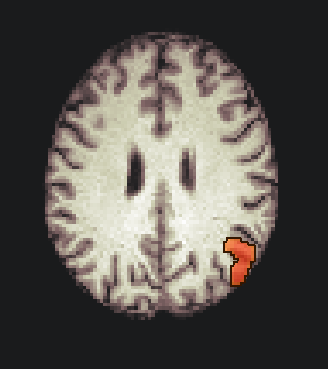


Left PL


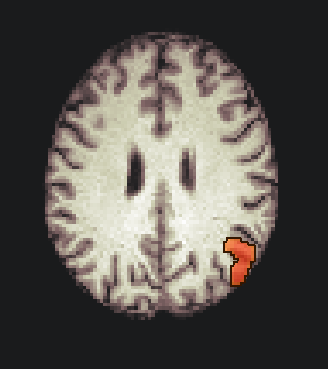


1. Salience Network

ACC


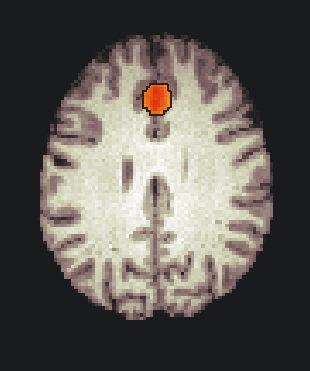


Left PPC


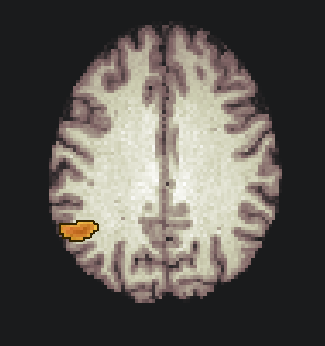


Right PPC


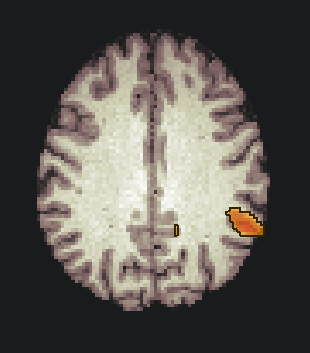


Right RPFC


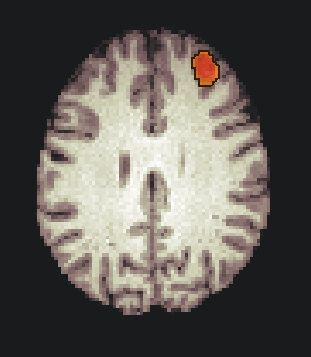


Left RPFC


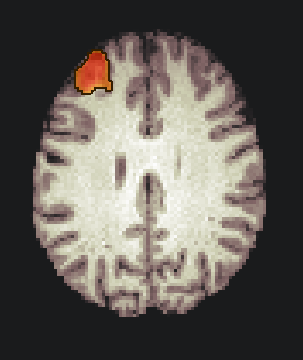


Left anterior insula


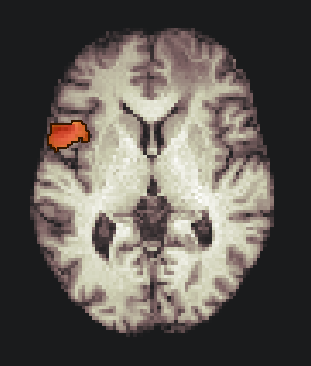


Right anterior insula


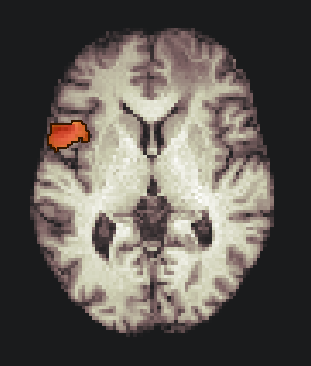

Supplement: Supplementary file 2 [file Supplementary_file_1.docx]
