# Supplementary material for: Reorganization of brain networks in olfactory groove meningioma patients: a pilot resting-state fMRI study
Source: Front Neurol. 2025 Aug 29;16:1644138. doi: 10.3389/fneur.2025.1644138 (PMC12425792; doi:10.3389/fneur.2025.1644138)
Supplement: Supplementary file 3 [file Table_1.docx]

***Supplementary Table 1.*** *Parameters of MRI sequences*

|  | ***T1WI*** | ***T2WI*** | ***FLAIR*** | ***BOLD-fMRI*** | ***TOF*** | ***DWI*** |
| --- | --- | --- | --- | --- | --- | --- |
| *Sequence type* | *3D TFE* | *3D TSE* | *3D TSE* | *2D* | *3D* | *2D EPI* |
| *Acquisition plane* | *Axial* | *Sagittal* | *Sagittal* | *Axial* | *Axial* | *Axial* |
| *TR (msec)* | *6.7* | *3500* | *4800* | *3000* | *23* | *3800* |
| *TE (msec)* | *2.98* | *300* | *340* | *30* | *3.45* | *90* |
| *Flip angle* | *8* | *90* | *90* | *90* | *18* | *90* |
| *Matrix* | *256х256* | *256х256* | *228x228* | *80x80* | *464x326* | *128x128* |
| *FOV (mm)* | *256* | *256* | *256* | *240* | *200* | *256* |
| *Slice thickness (mm)* | *1* | *1* | *1.2* | *3* | *0.8* | *4* |
| *N of slices* | *192* | *360* | *140* | *45* | *160* | *26* |
| *Inversion time (msec)* | */* | */* | *1650* | */* | */* | */* |
| *b values* | */* | */* | */* | */* | */* | *0, 500, 1000* |
| *Acquisition time*  *(min:sec)* | *6:11* | *5:19* | *4:24* | *10:00* | *4:57* | *2:05* |
