# Supplementary material for: Reorganization of brain networks in olfactory groove meningioma patients: a pilot resting-state fMRI study
Source: Front Neurol. 2025 Aug 29;16:1644138. doi: 10.3389/fneur.2025.1644138 (PMC12425792; doi:10.3389/fneur.2025.1644138)
Supplement: Supplementary file 4 [file Table_2.docx]

*Supplementary Table 2. The results of the GLM analysis examining the effects of morphometric data and clinical scores on functional connectivity within the OGMs group*

| **Network** | **Seed** | **Centre of cluster**  **(x; y; z)** | **Size of cluster (voxels)** | **T-value** | **p-FWE** | **Involved anatomical structures** |
| --- | --- | --- | --- | --- | --- | --- |
| ***Karnofsky performance scale (KPS)*** | | | | | | |
| *Default Mode Network (DMN)* | left PL | +12; -22; +62 | 2748 | + 4.5 | 0.031 | left and right precentral gyrus left, left superior frontal gyrus, right middle frontal gyrus |
| *Fronto-Parietal Network (FPN)* | right PPC | +28; -72; +26 | 4742 | - 5.6 | 0.003 | right lateral occipital, intracalcarine, cuneal, precuneous cortex, right lingual gyrus |
| *Salience network* | left anterior insula | +34; +12; +10 | 1972 | + 6.3 | 0.017 | right central opercular, insular, parietal operculum cortex, right Heschl's gyrus, right supramarginal gyrus |
|  | right anterior insula | +08; -30; +26 | 2645 | + 7.2 | 0.013 | cingulate gyri bilaterally |
| ***MoCA*** | | | | | | |
| *Default Mode Network (DMN)* | left PL | +10; -26; +66 | 3193 | + 6.3 | 0.02 | right precentral and postcentral gyri |
|  | MPFC | +38; +28; +12 | 3448 | - 6.8 | 0.008 | right frontal pole, right middle frontal gyrus |
| *Fronto-Parietal Network (FPN)* | left PPC | +34; -44; -10 | 2589 | - 6.9 | 0.03 | right lingual gyrus, right fusiform cortex |
|  | right PPC | +42; -62; +16 | 4111 | - 7.3 | 0.002 | right lateral occipital, precuneous, intracalcarine cortex, right superior parietal lobule, right angular gyrus |
| ***VAT*** | | | | | | |
| *Default Mode Network (DMN)* | MPFC | -4; -92; +28 | 2192 | + 4.9 | 0.038 | right and left occipital pole, right and left cuneal cortex |
| *Salience network* | left SMG | -12; +18; +70 | 2891 | + 5.1 | 0.029 | left frontal pole, left superior and middle frontal gyri, right paracingulate cortex and superior frontal gyrus |
| ***Peritumoral edema volume*** | | | | | | |
| *Salience network* | right anterior insula | -02; -40; +12 | 2344 | + 5.2 | 0.023 | precuneous cortex and posterior cingulate gyri bilaterally, right thalamus |

Abbreviations: MoCA – Monreal Cognitive Assessment, MPFC – medial prefrontal cortex; PCC – posterior cingulate cortex; PL – posterolateral cortex; PPC – posterior parietal cortex; ACC – anterior cingulate cortex, SMG – supramarginal gyrus; VAT – visual acuity test
